# Interference of skeleton photoperiod in circadian clock and photosynthetic efficiency of tea plant: in-depth analysis of mathematical model

**DOI:** 10.1093/hr/uhae226

**Published:** 2024-08-08

**Authors:** Zhi-Hang Hu, Ting Huang, Nan Zhang, Chen Chen, Kai-Xin Yang, Meng-Zhen Sun, Ni Yang, Yi Chen, Jian-Ping Tao, Hui Liu, Xing-Hui Li, Xuan Chen, Xiong You, Ai-Sheng Xiong, Jing Zhuang

**Affiliations:** Tea Science Research Institute, College of Horticulture, Nanjing Agricultural University, Nanjing, Jiangsu 210095, China; State Key Laboratory of Crop Genetics & Germplasm Enhancement and Utilization, Nanjing Agricultural University, Nanjing, Jiangsu 210095, China; State Key Laboratory of Crop Genetics & Germplasm Enhancement and Utilization, Nanjing Agricultural University, Nanjing, Jiangsu 210095, China; State Key Laboratory of Crop Genetics & Germplasm Enhancement and Utilization, Nanjing Agricultural University, Nanjing, Jiangsu 210095, China; Tea Science Research Institute, College of Horticulture, Nanjing Agricultural University, Nanjing, Jiangsu 210095, China; Tea Science Research Institute, College of Horticulture, Nanjing Agricultural University, Nanjing, Jiangsu 210095, China; Tea Science Research Institute, College of Horticulture, Nanjing Agricultural University, Nanjing, Jiangsu 210095, China; Tea Science Research Institute, College of Horticulture, Nanjing Agricultural University, Nanjing, Jiangsu 210095, China; State Key Laboratory of Crop Genetics & Germplasm Enhancement and Utilization, Nanjing Agricultural University, Nanjing, Jiangsu 210095, China; State Key Laboratory of Crop Genetics & Germplasm Enhancement and Utilization, Nanjing Agricultural University, Nanjing, Jiangsu 210095, China; Tea Science Research Institute, College of Horticulture, Nanjing Agricultural University, Nanjing, Jiangsu 210095, China; Tea Science Research Institute, College of Horticulture, Nanjing Agricultural University, Nanjing, Jiangsu 210095, China; College of Sciences, Nanjing Agricultural University, Nanjing, Jiangsu 210095, China; State Key Laboratory of Crop Genetics & Germplasm Enhancement and Utilization, Nanjing Agricultural University, Nanjing, Jiangsu 210095, China; Tea Science Research Institute, College of Horticulture, Nanjing Agricultural University, Nanjing, Jiangsu 210095, China

## Abstract

The circadian system of plants is a complex physiological mechanism, a biological process in which plants can adjust themselves according to the day and night cycle. To understand the effects of different photoperiods on the biological clock of tea plants, we analyzed the expression levels of core clock genes (*CCA1*, *PRR9*, *TOC1*, *ELF4*) and photosynthesis-related genes (*Lhcb*, *RbcS*, *atpA*) under normal light (light/dark = 12 h/12 h, 12L12D) and took the cost function defined by cycle and phase errors as the basic model parameter. In the continuous light environment (24 h light, 24L), the peak activity and cycle of key genes that control the biological clock and photosynthesis were delayed by 1–2 h. Under a skeleton photoperiod (6L6D, 3L3D), the expression profiles of clock genes and photosynthesis-related genes in tea plants were changed and stomatal opening showed a circadian rhythm. These observations suggest that a skeleton photoperiod may have an effect on the circadian rhythm, photosynthetic efficiency and stomatal regulation of tea plants. Our study and model analyzed the components of circadian rhythms under different photoperiodic pathways, and also revealed the underlying mechanisms of circadian regulation of photosynthesis in tea plants.

## Introduction

Tea (*Camellia sinensis* (L.) O. Kuntze), a perennial evergreen woody plant belonging to the *Camellia* genus in Theaceae, is extensively cultivated worldwide. As an economic crop, the total area of tea gardens in China reached 3.433 million hectares in 2023 [1]. Light played a crucial role in the growth and development of tea plants, influencing the morphology, developmental changes, and overall growth patterns. By perceiving light stimuli, plants could detect its presence and gather information regarding seasonal cues, such as day length, light directionality, and circadian clock interference [2].

Light intensity, light duration, and light quality are the key environmental factors affecting the growth and development of the tea plant, which together determine the metabolic state and physiological and developmental stages of tea plants [3–6]. Plants sensitively regulate their seasonal growth and flowering through photoperiodic signals. Wang and colleagues, by studying mutants with abnormal photoperiodic growth, revealed the regulatory mechanism of seasonal growth under long-day conditions [7]. The publication of tea genome papers provides a lot of help for the study of tea molecular biology. Till now, research on growth, development, and secondary metabolism of the tea plant has mainly focused on theanine biosynthesis [8, 9], anthocyanin biosynthesis [10], flavonoid biosynthesis [11], polyphenol biosynthesis [12], and responses to abiotic stress [13, 14]. Although photoperiod and circadian rhythm have potential impacts on tea plants, there has been relatively little research on their roles in tea plants [15].

The biological clock is an exquisite internal timing mechanism within plants and animals, recording the developmental patterns formed by organisms during the process of evolution. This ‘invisible clock’ demonstrates how nature shapes plants to adapt to the needs of long-term survival through phenomena such as tree rings, leaf growth, fruit maturation, and abscission [16, 17]. In plants, the biological clock is primarily composed of three key parts: the output pathway, the central oscillator, and the input pathway, which work in concert to respond to environmental changes [18, 19]. Light and temperature are the two critical zeitgebers for plant circadian rhythms, affecting the physiological and behavioral rhythms of plants [20, 21].

As our understanding of the biological clock mechanism has deepened, we have learned that the core circadian rhythm circuit has evolved from a single negative feedback pattern to a complex system that includes multiple negative feedback loops, involving transcription–translation feedback loops (TTFLs) [22–24]. Specifically, the core biological clock is composed of dawn genes *CIRCADIAN CLOCK ASSOCIATED 1* (*CCA1*) and *LATE ELONGATED HYPOCOTYL* (*LHY*), morning genes *PSEUDO-RESPONSE REGULATORS 9*, *7*, and *5* (*PRR9*, *7*, *5*), dusk gene *TIMING OF CAB1* (*TOC1*), and evening genes *EARLY FLOWERING 4* (*ELF4*) and *LUX ARRHYTHMO* (*LUX*) [25–27]. The circadian rhythm model has undergone a process from being simple [28] to complex [29], and then to being compact [30]. In this model, these components serve as repressors to suppress other transcription factors at different times of the day. Although a depreciation-based system could oscillate, its robustness could be enhanced by integrating a positive feedback loop. At the beginning of the day, a new clockprotein, LIGHT-REGULATED WD1 (LWD1), is involved in photoperiod control; LWD1 activates *CCA1* [31], *PRR9* [32], and *TOC1* [33] to form a positive feedback loop..

The biological clock regulates physiological processes such as photosynthesis, stomatal switching, and leaf movement in plants through its output pathways, which are crucial for plant growth and development [34, 35]. Photosynthesis is the process of capturing light energy and converting it into chemical energy. The *LHCA* and *LHCB* gene families encode chlorophyll *a*/*b*-binding polypeptides for photosystems I and II, as well as genes involved in the biosynthesis of chlorophyll and the *RUBISCO SMALL SUBUNIT* (*RBcS*), which participates in carbon fixation [36–40]. Therefore, we selected photosynthesis-related genes *Lhcb1*, *RbcS1*, and *atpA* as the output variables of the biological clock for photosynthesis. Locke *et al*. found that *LHY* and *CCA1* could rapidly respond to environmental light changes, regulate the expression of specific genes, GI, at night, and constructed the first differential equation model [28]. Ebenhoh *et al*. proposed an ordinary differential equation (ODE) model for the dynamic regulation of photosynthesis in eukaryotes to reproduce the basic fluorescence characteristics of short-term light adaptation [41]. Using a computational approach to circadian-clock-controlled photosynthesis, Huang and her colleagues found that low light intensity prolonged the photosynthesis cycle and delayed the peak time of photosynthesis in tomato. Through experiments and mathematical model predictions, it was found that, under low light, the plant’s biological clock and photosynthetic parameters would shift backward by 1–2 h, while under high light they would shift forward by 1 h [42].

In this study, we investigated photosynthetic efficiency under different photoperiods through the biological clock network and developed a computational mathematical model. The light-induced circadian clock sensed different photoperiod inputs, leading to changes in the endogenous rhythm of plants, and ultimately affecting the expression of photosynthesis-related genes and changes in photosynthetic parameters. The changes in stomata under different photoperiods were also measured by constructing the non-linear relationship among the expression profiles of the core genes of the biological clock and photosynthesis-related genes, photosynthetic parameters, and changes in stomatal aperture. Predicting the effects of different photoperiods on the daily rhythm of tea plants can improve understanding of how tea plants adapt to natural photoperiod fluctuations through their internal biological clock mechanisms.

## Results

### Rhythmic expression of core clock gene under four kinds of photoperiod in tea plants

In order to study the rhythmic expression profiles of tea plant core clock genes under different photoperiods, four photoperiod treatments were applied to tea plants at constant temperature (Supplementary Data Fig. S1). According to the H2023 model (The model of Huangting-2023) [42], we used nine differential equations (equations S1–S9) that incorporate transcriptional levels, protein abundances and the activated proportion of photosensitive proteins, which were defined as MCL, CL, MP97, P97, MP51, P51, MEL, EL, and P (Supplementary Data Table S2).

Tea plants ‘Baiye 1’ were placed in a light incubator under 12 h light/12 h dark (12L12D) for 1 week, and then transferred to four different photoperiod incubators, comprising long day (12L12D), constant light (24L), long skeleton photoperiod (6L6D) and short skeleton photoperiod (3L3D) (Supplementary Data Fig. S1).

First, we drew the data to fit LWD1 expression under 24L, which was fitted as a second-order Fourier series:


\begin{align*} \left[\mathrm{LWD}\right]&=0.45\sin \left(\frac{2\pi }{26}t\right)+0.4\cos \left(\frac{2\pi }{26}t\right)+0.48\sin \left(\frac{2\pi }{26}2t\right)\\&\quad+0.1\cos \left(\frac{2\pi }{26}2t\right)+1.52. \end{align*}

Moreover, we calculated the mean square error (MSE), where ${y}_i$ and ${\tilde{y}}_i$ denote simulated values and experimental values, respectively:


$$ \textrm{MSE}=\sum_i{\left({y}_i-{\tilde{y}}_i\right)}^2=30.0285. $$

Second, the parameters of the model were evaluated by minimizing the cost function. In consequence, we obtained an optimal parameter set, called the basal parameter set. Third, the function equations for LWD under other photoperiodic conditions were obtained by the same fitting method of Fourier series and the MSEs. Furthermore, the expression predictions of circadian oscillators under the other three photoperiods were made based on the basal parameter set in the following step by changing the light input function. Under the 12L12D photoperiod, we saw that *CsCL* peaks at dawn (T0). *CsP51* peaks at dusk (T12), *P97* peaks at T6–T9, and *CsEL* peaks at T12–T15. The expression of the core circadian oscillator was predicted by changing the light/dark input (24L, 6L6D, 3L3D) based on the circadian rhythm model of the tea plant under the conventional cycle (12L12D), and it was found that the prediction results of the model were basically consistent with the experimental values.

**Figure 1:**
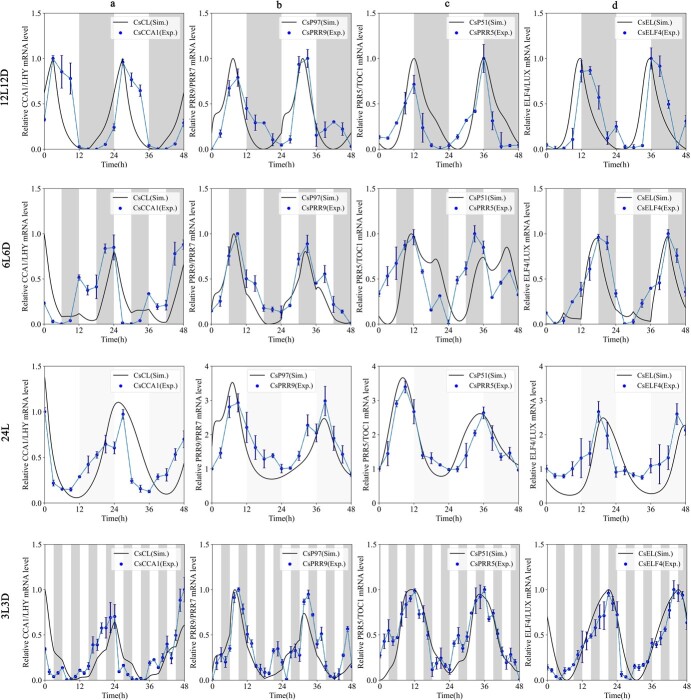
Dynamics validation of the core circadian elements under four photoperiods. **a**  *CCA1* mRNA expression profile in relation to *CL* level simulation. **b**  *PRR9* mRNA expression profile in relation to *P97* level simulation. **c**  *TOC1* mRNA expression profile in relation to *P51* level simulation. **d**  *ELF4* mRNA expression profile in relation to *EL* level simulation. Time courses of clock elements were predicted (solid lines) and compared with experimental data (blue dots), which were quantified using RT–qPCR. Gray bands represent objective darkness. *n* = 3 biologically independent samples, and the results represent mean ± standard error.

Under the photoperiod of 6L6D, we found that the fluctuating pattern of core clock genes displayed minimal discernible variations, but gene expression was changed at different time points due to the influence of light. As shown in Fig. 1, the peak time of the four pairs of genes moved back roughly 1–2 h, and gene expression became unstable in the change of light and darkness. The cycle of *CCA1* and *ELF4* transcripts decreased during the day, while the cycle of *PRR5* increased by half when night came. The period of *PRR9* was the same as that at 12L12D. Under constant light, all four pairs of clock genes in the tea plant were rhythmical. Under the skeleton photoperiod (3L3D), *CCA1*/*LHY* (a typical gene usually expressed in the early morning) showed a shortened period and unstable expression during a shorter photoperiod. Nevertheless, *PRR9/PRR5* (*P97*) and *PRR5*/*TOC1* (*P51*) genes maintained a certain rhythm at the transcriptional level, and their expression patterns were relatively stable overall. The *ELF4/LUX* gene was basically consistent with the model prediction, although its expression cycle deviated from normal at some specific time points.

### Clock control of photosynthetic gene dynamics under different photoperiods

**Figure 2:**
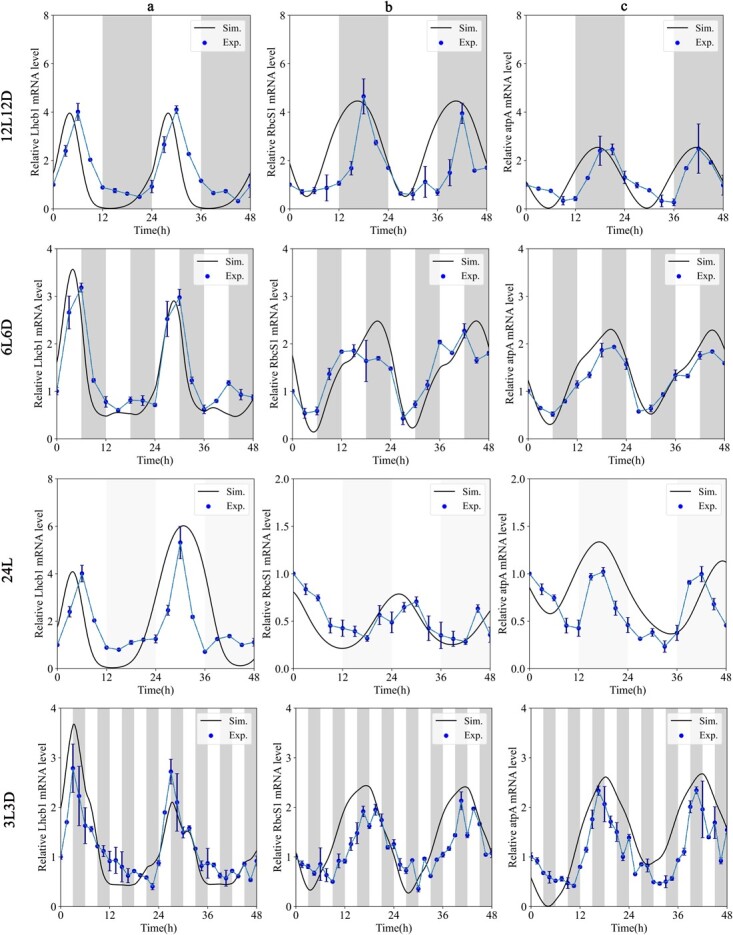
The circadian clock controlled the dynamic behavior of photosynthetic genes in tea plants under four photoperiodic treatments. Simulated expression of *Lhcb1* (**a**), *RcbS1* (**b**), and *atpA* (**c**). Time courses of the clock elements were predicted (solid lines) and compared with experimental data (blue dots), which were quantified using RT–qPCR. Gray bands represent objective darkness. *n* = 3 biologically independent samples, and the results represent mean ± standard error.

With reference to the H2023 model [42], we conducted expression analysis of *Lhcb1*, *RbcS1*, and *atpA* and constructed mathematical models. To investigate the effect of the skeleton light cycle on photosynthesis, we quantified the transcription levels of these photosynthetic genes against the regular light cycle and different photoperiod conditions (Fig. 2). The circadian regulation function was crucial to the photosynthesis of tea plants, and the dynamics of *Lhcb1* and *RbcS1* under the control of the circadian rhythm still followed the H2023 model.

Under the 12L12D photoperiod, the expression level of the *Lhcb1* gene was high in the day, low in the night, and peaked at the T6 stage. In contrast, the *RbcS1* and *atpA* genes had high expression levels in night and low expression in the day, and reached a peak at T18. In the 6L6D stage, the expression level of the *CsLhcb* gene was significantly reduced in the T6–T12 stage, indicating that the expression of this gene was inhibited under the dark cycle. However, when the tea plant received the light signal again, the *Lhcb* gene expression level became higher again. The *RbcS1* and *atpA* genes with high expression levels at night entered the dark cycle in advance, but their expression levels did not increase. The trends for the *CsLhcb* gene and *atpA* gene were basically the same as those for 12L12D under constant light, but the cycle of the *RbcS1* gene seemed to advance. The difference analysis demonstrated that under different photoperiods the expression waveforms were significantly different, especially in skeleton cycles of light and dark (6L6D and 3L3D). We calculated the period under 24L and 12L12D conditions, which showed that the periods of photosynthetic genes performed at 25.85 ± 0.18 and 24.11 ± 0.13 h, respectively. The period extension and phase delay of 1–2 h under 24L were similar to those in *Arabidopsis* [43]. Under 6L6D and 3L3D conditions, the simulated curves showed one or two multiple shoulders between two peaks. These results indicated that skeleton photoperiods may be conducive to altering the oscillating curve, period, and phase of photosynthetic genes, consistent with results for the circadian rhythm.

### Changes in photosynthetic parameters of tea plant under different photoperiods

We modeled photosynthetic parameters using *CCA1*, *Lhcb1*, *RbcS1*, and *atpA* to represent photosynthetic efficiency (*P*_i_). The defined equation describes the kinetics of the system:


\begin{align*} {\mathrm{P}}_{\mathrm{i}}={\mathrm{\alpha}}_{\mathrm{i}}+\frac{{\left[\mathrm{CL}\right]}^2}{{\mathrm{K}}_{\mathrm{i}1}^2+{\left[\mathrm{CL}\right]}^2}+\sum_{\mathrm{j}}\frac{{\left[{\mathrm{X}}_{\mathrm{j}}\right]}^2}{{\mathrm{H}}_{\mathrm{i}\mathrm{j}}^2+{\left[{\mathrm{X}}_{\mathrm{j}}\right]}^2} \end{align*}

In this context, [CL] denotes *CCA1*, while [*X_j_*] (*j* = 1, 2, 3) represents the mRNA levels of *Lhcb1*, *RbcS1*, and *atpA*. Other parameters were elucidated as follows: *i* signifies the four photosynthetic parameters *P*_n_, *G*_s_, *C*_i_, and *T*_r_; α stands for the fundamental photosynthetic rate. For a specific photosynthetic parameter *i*, *K*_*i*1_ and *H_ij_* denote the *CCA1* activation and the facilitation intensity of *Lhcb1*, *RbcS1*, and *atpA* on *P_i_*, respectively. These dynamic parameters exhibited variances across distinct photosynthetic rate models.

**Figure 3:**
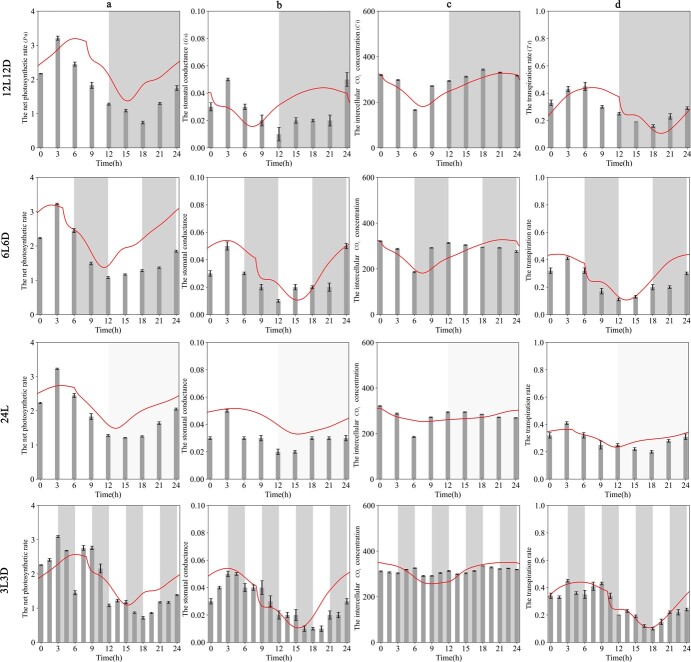
Changes in photosynthetic parameters of tea leaves under four photoperiodic treatments. **a** Net photosynthetic rate (*P*_n_). **b** Stomatal conductance (*G*_s_). **c** Intercellular CO_2_ concentration (*C*_i_). **d** Transpiration rate (*T*_r_). Red solid lines denote photosynthetic simulations. Gray bands represent objective darkness. *n* = 3 biologically independent samples, and the results represent mean ± standard error.

Compared with the control photocycle (12L12D), the *P*_n_ under continuous illumination was basically the same, reaching a peak value of 3.23 μmol m^−2^ s^−1^ in the T3 stage, and *P*_n_ was higher in the T15–T24 stage than in the 12L12D stage. Both peak position and peak width decreased (24L). At 6L6D, *P*_n_ showed a lower biological peak in advance. Especially at 3L3D, the number of peaks increased and the *P*_n_ concentration changed irregularly (Fig. 3).

There was no significant difference observed in stomatal conductance (*G*_s_) between the 12L12D condition and the 24L condition (Fig. 3b), albeit a slight rise from T12 to T24. This suggested an augmentation in stomatal conductance under continuous light. However, under the 6L6D and 3L3D conditions the waveform and rhythm also did not obviously change from the normal photoperiod of 12L12D. This shows that the *G*_s_ value of the tea plant had little effect under different photoperiods.

Compared with 12L12D, the trend of change in *C*_i_ under various photoperiodic treatments exhibited a consistent pattern, with concentrations peaking during the nocturnal period. Under continuous illumination, the *C*_i_ levels moderated during the interval from T12 to T24. The *T*_r_ waveform was similar to that of *P*_n_ and *G*_s_. Under 12L12D conditions, the peak appeared in the daytime for ~3 h and remained low at night. The value of *T*_r_ gradually increased again before approaching the next light cycle. The concentration and curve type of *T*_r_ in the same skeleton period were consistent with *G*_s_. There were many small spikes on the *T*_r_ curve at 3L3D, indicating a disruption in rhythm.

### Changes in stomatal opening in tea plants under different photoperiods

In order to understood the stomatal opening degree of tea leaves during the day, we detected and analyzed tea leaves under four photoperiods. It was found that stomatal opening of tea leaves was different at different time points (Supplementary Data Table S1). In the 12L12D cycle, the tea leaves opened slowly from 0 to 6 h, and the opening gradually became larger, and reached the peak value at ZT6, which was 58.80 μm^2^. Then the tea plant took a short lunch break and the stomata gradually narrowed with the arrival of the night, reaching a low point at ZT15 with a stoma size of only 34.29 μm^2^ (Supplementary Data Fig. S2A, Table 1). Tea leaves in the 6L6D cycle had obvious changes compared with those in the T1 treatment group. In the ZT9 and ZT12 stages the stomatal size of tea leaves shrank, while in the ZT15 and ZT18 stages the stomatal opening of tea leaves increased significantly (Supplementary Data Fig. S2B).

The trend of stomata opening in tea leaves under continuous light was similar to that in constant light periods, but in the ZT15–ZT24 period the stomata of tea leaves did not shrink due to long-term light. The first three treatments all peaked at ZT6 (Supplementary Data Fig. S2). Under the most complex skeletal photoperiod (3L3D) we found that the stomata of tea leaves open in response to light production and close under dark conditions. The maximum value of 56.20 μm^2^ was reached in the T3 stage. Unstable light conditions could lead to irregular stomatal opening (Supplementary Data Fig. S3).

## Discussion

Photoperiod is one of the very important environmental factors in the growth and development of plants, and has a significant impact on the physiological and developmental processes of plants. This study simulated different daylight cycles by applying four different types of photoperiod treatments (12L12D, 24L, 6L6D, 3L3D) to investigate the adaptability and flexibility of the biological clock in tea plants under atypical light conditions. This design can reveal the effect of the sunshine cycle on the biological clock and photosynthesis of the tea plant, and then provide a scientific basis for the cultivation management and breeding of tea plants. Li *et al*. increased the absorption and accumulation of nitrogen and phosphorus, especially potassium, in cucumber seedlings by extending the light time [44]. The accumulation of micronutrients in cucumber seedlings also increased with extension of the light supplement time. He *et al*. studied the effects of three different photoperiods on tomato plants, and found that under short-day conditions (7 h of light/5 h of dark and 3.5 h of light/2.5 h of dark), tomato leaves showed symptoms of green deficiency [45]. Kang *et al*. [46] further investigated the effects of light intensity and photoperiod on the growth and morphology of lettuce, and the results showed that high light intensity [290 μmol m^−2^ s^−1^ PPFD (photosynthetically active radiation density)] combined with a short photoperiod (6 h light/2 h dark) was beneficial to the overall growth and development of lettuce. Medium light intensity (230 or 260 μmol m^−2^ s^−1^ PPFD) combined with a longer photoperiod (18 h light/6 h dark and 9 h light/3 h dark) was more conducive to plant growth and improved photosynthetic capacity. By studying the combination of multiple environmental factors such as light, light quality, and temperature, the optimal growth conditions for tea plants were determined, thereby reducing experimental time and resource consumption.

**Figure 4:**
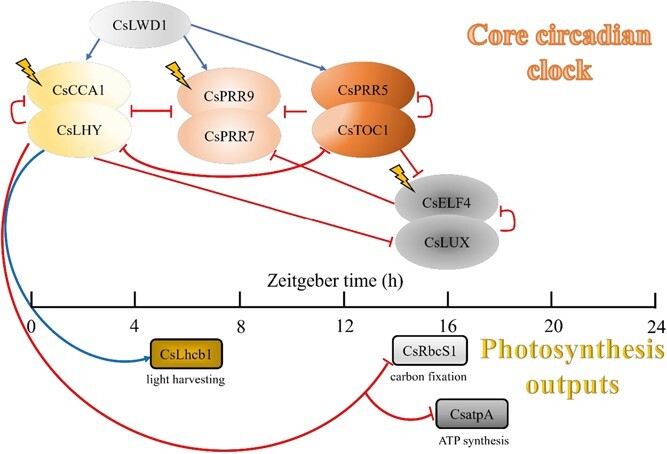
Schematic illustration of the circadian clock that regulates the photosynthetic production of the tea plant. A computational model was developed to study the circadian clock control of photosynthesis using a regulatory network composed of the light-sensitive protein P, core oscillator, and photosynthetic metabolic outputs. In this model, solid lines blunt at the terminal ends represent genes exerting inhibitory effects in a negative feedback loop, while arrows indicate genes that have an activating role in the regulatory network. A lightning symbol denotes transcription activation of specific genes by light.

In higher plants, the core oscillator of the circadian clock consisted mainly of four pairs and three individual clock genes (Fig. 4) [42]. Through our study, we found that under a normal photoperiod (12L12D) the four pairs of clock genes showed normal oscillatory rhythms in tea plants. This was consistent with previous studies [47, 48]. The circadian rhythm of the tea plant changed with the change in photoperiod. Under constant light, all four pairs of clock genes in the tea plant were rhythmical. At 6L6D, the biological clock of the tea plant showed different degrees of periodic changes. The cycle of CCA1 and ELF4 transcripts decreased, while the cycle of PRR5 increased by half. The period of *PRR9* was the same as that at 12L12D. Under the skeleton photoperiod (3L3D), the expression of tea plant circadian rhythm genes was unstable. *CL* (*CCA1*/*LHY*), as a typical early-morning gene, had a halving of its cycle and unstable gene expression when the photocycle became shorter, and *EL* (*ELF4*/*LUX*) had a large arrhythmia. Recent studies have shown that under continuous light conditions, the pattern of autophagy rhythms differs from the normal light/dark cycle, specifically manifested by advanced phase and reduced amplitude. In addition, the mutation of the transcription factor LUX eliminated the autophagy rhythm under constant light (24L) conditions and led to increased amplitude under light/dark cycle (12L12D) conditions [49].

The transcription levels of *P97* (*PRR9/PRR5*) and *P51* (*PRR5/TOC1*) were rhythmically stable in general, although there was a large cycle deviation at some time points. According to the above results, the circadian clock model of tea plant will show different patterns under different photoperiods. Shortening the photoperiod to half of the original will halve the cycle of some specific clock genes, and further shortening the photoperiod may completely disrupt the rhythm of the biological clock, causing it to lose its normal rhythm. The timings of circadian gene expression and various regulatory relationships within the core oscillator were critical for maintaining circadian rhythms. Circadian clock core oscillators form highly linked regulatory networks with other circadian clock components, widely influencing plant signaling and metabolic pathways [50].

**Table 1:** Stomatal aperture of tea leaves at different times.

**Time (h)**	**Photoperiod**			
	**12L12D**	**6L6D**	**24L**	**3L3D**
0	44.46 ± 0.42^c^	45.18 ± 0.51^b^	43.37 ± 1.07^d^	43.62 ± 1.39^ef^
1.5				48.14 ± 0.27^bc^
3	56.86 ± 1.20^ab^	58.24 ± 1.60^a^	55.26 ± 0.93^ab^	56.20 ± 1.04^a^
4.5				49.27 ± 0.86^b^
6	58.80 ± 0.80^a^	58.72 ± 0.69^a^	58.86 ± 2.46^a^	43.96 ± 0.80^def^
7.5				46.05 ± 0.82^cde^
9	54.65 ± 0.98^b^	38.62 ± 0.88^d^	56.26 ± 0.49^ab^	50.41 ± 1.71^b^
10.5				39.22 ± 0.87^h^
12	55.60 ± 1.22^b^	38.43 ± 1.62^d^	52.46 ± 2.66^bc^	40.47 ± 1.54^gh^
13.5				46.58 ± 0.32 ^cd^
15	34.29 ± 2.19^e^	44.24 ± 1.48^b^	49.20 ± 1.51^c^	45.85 ± 1.20^cde^
16.5				44.55 ± 1.34^def^
18	35.26 ± 0.50^e^	42.97 ± 0.62^bc^	45.60 ± 0.98^d^	40.21 ± 1.92^h^
19.5				44.54 ± 0.78^def^
21	41.77 ± 0.32^d^	41.67 ± 0.86^c^	50.27 ± 1.90^b^	45.48 ± 0.81^def^
22.5				39.24 ± 1.53^h^
24	45.72 ± 1.44^c^	43.72 ± 0.83^bc^	52.52 ± 1.53^bc^	42.83 ± 1.31^fg^

Different letters indicate statistically significant differences (*P*<0.05)

Photosynthesis is the key cellular process that takes place at a specific time of day and has been reported to be controlled by a circadian clock [51, 52]. The *LHCA* and *LHCB* gene families encode chlorophyll *a*/*b*-binding polypeptides for photosystems I and II, as well as genes involved in the biosynthesis of chlorophyll and *RUBISCO SMALL SUBUNIT* (*RBCS*), participating in carbon fixation [53]. The expression profiles of these photosynthesis-related genes participating in light harvesting, carbon fixation, and ATP production are under clock control [42]. The circadian clock is thought to give plants an advantage, but the exact nature of this advantage has been unclear. Studies have shown that in *Arabidopsis* there is a significant photosynthetic advantage if the circadian cycle is correctly matched to the external light–dark cycle. In both long- and short-day conditions, plants whose circadian cycles are adapted to their environment contain more chlorophyll, fix more carbon, grow faster, and survive better. Conversely, if a plant’s biological clock cycle does not match its environment, it is less effective [51]. Our results showed that when tea plants were exposed to light treatment for a long time and consistently with the environmental cycle of the biological clock, the photosynthetic utilization efficiency and the expression of photosynthetic genes should become higher. When tea plants were exposed to unstable light conditions (6L6D, 3L3D), the photosynthetic utilization efficiency and stomatal aperture of the plants were different from that under the normal photoperiod. From ZT3 to ZT12, stomatal opening under 12L12D and LL conditions was generally significantly higher than that under 6L6D and 3L3D conditions. The results indicated that the skeleton photoperiod had a significant effect on the circadian system of the tea plant, resulting in irregularity of the circadian clock, reducing the efficiency of photosynthesis and stomatal opening. In tomato, the circadian cycle changes as light intensity changes. Under low light conditions, the peak time of the clock and photosynthetic genes shifted backwards by 1–2 h, and the cycle extended by roughly the same length [42]. A photoperiodic deviation from the 24 h cycle negatively affects the growth of tomato plants, which are significantly damaged by prolonged exposure to constant light [54]. Hearn *et al*. showed how dynamic periodic adjustment of circadian oscillators could help establish the correct phase relationship with the environment [55]. Photoperiodic changes also affect plant responses to environmental stress, such as drought, cold, and saline–alkali stress. By adjusting their circadian rhythm, plants are better prepared to deal with these challenges. Kusakina *et al*. found that leaf movement matched the periodic activity of *CCA1* and *LHY* genes only at suitable temperatures, suggesting a regulatory role for these genes by the circadian clock. However, under other temperature conditions, this regulatory effect was weakened, which was crucial to the adaptability of plant growth, which also provided a reference for further understanding of the effect of cycle shortening on plant growth performance [56]. The widespread effects of circadian systems on crops at this stage suggest that future food production may be improved by altering the circadian rhythm, designing the timing of transgenic expression, and applying agricultural treatments at the most efficient times of the day [57].

In addition, an important strength of our research lies in the development and application of a mathematical model that captures the complex dynamics of the circadian system of the tea plant. The model predicted the behavior of the system under different photoperiods and also provided a framework for understanding the underlying mechanisms of circadian regulation in tea plants. For example, adjusting the lighting of a tea garden or controlling the growing environment based on an understanding of the circadian rhythm can improve the yield and quality of tea. In addition, the model can be used as a predictive tool to develop strategies to mitigate the effects of environmental stress on tea plants by timing agronomic interventions according to day and night cycles.

In this study, we aimed to investigate the adaptability and flexibility of the circadian rhythm in tea plants by subjecting them to four non-standard light conditions in a photoperiod (24 h). We sought to gain insights into how tea plants adjust their physiological activities in response to different light regimes, and to enhance our understanding of how photoperiod regulation impacts the growth of tea plants. We plan to continue with the long-day (16L8D) or short-day (8L16D) experimental designs. These studies closely simulate the natural light cycle that tea plants experience in their native habitat, providing valuable information for biological research, cultivation management, and breeding of tea plants. Then, we will also treat tea plants under different light qualities (red, purple, blue) in more photoperiods (48 h or more). In the future, the biological clock of the tea plant can be optimized by adjusting the cultivation environment of the tea plant, such as the light cycle and intensity, so as to improve photosynthetic efficiency and tea yield and quality. Later, by further studying the function of tea plant clock genes, new breeding strategies can be developed, and tea plant varieties adapted to specific light conditions can be selected or designed to enhance their adaptability and stress resistance to environmental changes. The research can be extended to other plants to explore the adaptation mechanism of different plants to changes in the sunshine cycle, and provide scientific guidance and technical support for crop production against the background of global climate change. The obtained experimental data provide more information for further research on the mechanism of the tea plant circadian rhythm. Till now, there have been relatively few studies on the biological clock and photoperiod of tea plants. These studies are significant for understanding the mechanism of tea growth and development regulated by the circadian rhythm.

### Conclusions

Photoperiod has a significant effect on the circadian rhythm of tea plants. It was found that the expression patterns, cycles, and phase errors of core clock genes (*CL*, *P97*, *P51*, *EL*) and photosynthetic genes (*Lhcb*, *RbcS*, *atpA*) of tea plants changed under different photoperiodic conditions. In the continuous light environment (light = 24 h, 24L), the peak activity and cycle of key genes that control the biological clock and photosynthesis were delayed by 1–2 h. This suggested that constant light can lead to an adjustment of the circadian clock, which in turn affects photosynthesis. Under the skeleton photoperiods (6L6D and 3L3D), our study observed alterations in the expression patterns of core circadian genes and photosynthesis-related genes, as well as changes in photosynthetic parameters and stomatal opening. These findings suggest that such photoperiods may influence the circadian regulation of physiological processes in tea plants. This indicates that extreme changes in the photoperiod will interfere with the circadian rhythm of the tea plant and affect its growth and development.

Through mathematical model analysis, the study revealed the potential mechanism of the tea plant’s diurnal regulation of photosynthesis. The model predicted the variation trend of photosynthetic parameters under different photoperiods, and was consistent with the experimental data, which verified the accuracy of the model. Circadian oscillators played an important role in the photosynthesis of tea plants under different photoperiods. The regularity or irregularity of the circadian system may lead to the enhancement or disturbance of gene expression, affecting plant growth and development and phenotype. Dynamic modeling helps to increase the speed of crop breeding by studying the combination of multiple environmental factors such as light, light quality, and temperature to determine the optimal growth conditions, thereby reducing experimental time and resource consumption. In summary, this study discusses the influence of different photoperiods on the circadian rhythm and photosynthesis of tea plants through experimental and mathematical model analysis, which provided a potential scientific basis for tea plant cultivation and production management.

## Materials and methods

### Plant material and growth conditions

Cutting seedlings of 2-year-old ‘Baiye 1’ plants were cultivated in the State Key Laboratory of Crop Genetics & Germplasm Enhancement and Utilization, Nanjing Agricultural University (Nanjing, China, 188.84°E, 32.04°N). Tea seedlings with healthy growth were selected in a light incubator (temperature 25°C, photocycle 12 h/12 h, light intensity 240 μmol m^−2^ s^−1^, humidity 70 ± 5%). Tea seedlings were planted in sandy loam with good drainage, organic matter content of 1–2% or more, and pH = 6.0. After 1 week of cultivation, tea seedlings were placed in four light incubators with different photoperiods, i.e. long day (12 h light/12 h dark, 12L12D), constant light (24L) and two skeleton photoperiods (6L6D and 3L3D), temperature 25°C, light intensity 240 μmol m^−2^ s^−1^, and humidity 70 ± 5% (Table 2). All four treatments started sampling at 9 a.m. and the initial time was recorded as 0 h. Healthy tea seedlings were selected and one bud and two leaves were extracted every 3 h in the 12L12D, 24L, and 6L6D cycles. As the 3L3D cycle was special, we selected healthy tea seedlings every 1.5 h, picked one bud and two leaves, wrapped them with tin foil, quickly froze them with liquid nitrogen, and stored them at −80°C for subsequent experiments. Three biological replicates were performed for each sample.

**Table 2:** Different photoperiod treatment test schemes.

**Group**	**Time frame**															
	**ZT 0–** **ZT 1.5**	**ZT 1.5–** **ZT 3**	**ZT 3–** **ZT 4.5**	**ZT 4.5–** **ZT 6**	**ZT 6–** **ZT 7.5**	**ZT 7.5–** **ZT 9**	**ZT 9–** **ZT 10.5**	**ZT 10.5–** **ZT 12**	**ZT 12–** **ZT 13.5**	**ZT 13.5–** **ZT 15**	**ZT 15–** **ZT 16.5**	**ZT 16.5–** **ZT 18**	**ZT 18–** **ZT 19.5**	**ZT 19.5–** **ZT 21**	**ZT 21-** **ZT 22.5**	**ZT 22.5-** **ZT 24**
12L12D	12 h (09:00–21:00)							12 h (21:00–^+1^09:00)								
6L6D	6 h (09:00–15:00)			6 h (15:00–21:00)				6 h (21:00–^+1^03:00)				6 h (^+1^03:00–^+1^09:00)				
LL	24 h (09:00–^+1^09:00)															
3L3D	1.5 h (09:00–10:30)	1.5 h (10:30–12:00)	1.5 h (12:00–13:30)	1.5 h (13:30–15:00)	1.5 h (15:00–16:30)	1.5 h (16:30–18:00)	1.5 h (18:00–19:30)	1.5 h (19:30–21:00)	1.5 h (21:00–22:30)	1.5 h (22:30–24:00)	1.5 h (24:00–01:30)	1.5 h (01:30–03:00)	1.5 h (03:00–04:30)	1.5 h (04:30–06:00)	1.5 h (06:00–07:30)	1.5 h (07:30–09:00)

ZT xx represents the processing time, where ZT 0 and ZT 24 respectively represent the beginning and end of a day, the white part of the table represents light processing, the gray shading dark processing, and xx h (xx:xx–xx:xx) is the total duration of the light (dark) processing stage and the corresponding processing time (Beijing time). The superscript ^+1^ indicates the time of the next day. The first three groups were tested every 3 h for a total of 9 times, and the fourth group was tested every 1.5 h for a total of 17 times.

### Model description and construction

Based on the circadian rhythm pathway in *C. sinensis* [58], we redrew the schematic network (Fig. 4). On account of the absence of upstream genes regulating LWD1, we constructed periodic functions (Fourier series) of LWD1 concentrations under four different photoperiods to fit the experimental data. The model contained 15 variables (Supplementary Data Table S2) and 56 parameters (Supplementary Data Tables S3 and S4), including nine circadian oscillator variables (equations S1–S9) and six photosynthetic output variables (equations S10–S15; see Supplementary Material File).

In the modeling, each variable consisted of the corresponding concentration variations of mRNA and protein. The reactions included transcription, translation, and degradation. The red and blue lines in Fig. 4 denote inhibitions and activations of transcription, modeled by the decreasing ($\frac{1}{1+{\left(\frac{X}{K}\right)}^2}$) and increasing ($\frac{{\left(\frac{X}{K}\right)}^2}{1+{\left(\frac{X}{K}\right)}^2}$) Hill functions, respectively. The translation and degradation reaction modeling followed the mass action law (linear function) [30, 42, 59]. The input photoperiodic cues provided by light and darkness were represented by the functions $L(t)$ and $D(t)$ in the kinetic equations. The values of $L(t)=1$, $D(t)=0$ or $L(t)=0$, $D(t)=1$ denoted light switching on or off, respectively. The light and dark signals are transduced by photosensitive protein P to regulate the level variations of the circadian clock. In the circadian-clock-controlled photosynthesis pathway, the transcriptional levels of photosynthetic genes (*Lhcb1*, *RbcS1*, *atpA*) regulated by the core circadian element CCA1 perform as outputs in the circadian clock network. The model parameters associated with the core circadian clock remained invariant as in the previous model [59] and the parameters associated with LWD1 functions under different photoperiods were obtained by minimizing mean square errors between the simulations of Fourier series and experimental data. In addition, the parameters related to photosynthetic outputs (equations S10–S15) were estimated by minimizing the cost function [$\delta =\sum_{i=G}{\left({y}_i-\overline{y_i}\right)}^2$], where $G$ denotes the photosynthetic genes defined by the differences between the simulations ($y$) and the corresponding quantified transcription levels ($\overline{y}$) under 24L. Photoperiods were divided into a control group for parameter estimation (constant light, 24L) and a predictive group for verification (12L12D, 6L6D, 3L3D for one cycle).

### Parameter optimization

Model development is followed by model calibration, in which experimental data are often used to estimate the kinetic parameters. Some parameters can be simply adopted from a published paper. Others can be determined using some databases or experimental quantifications by oneself that store comprehensive information on biochemical reactions and their kinetic properties.

In general, experimental data are the temporal series of gene expression, containing the features of the model components, such as period, amplitude, phase, etc. This motivated us to construct a cost function to quantitatively assess the goodness of fit of our solution to qualitative features present in the experimental data. The simple cost function is defined by


$$ \delta =\sum_{i=G}{\left({y}_i-\overline{y_i}\right)}^2 $$

where $G$ denotes the photosynthetic genes *Lhcb1*, *RbcS1*, and *atpA*, and $y$ and $\overline{y}$ indicate the model simulations and experimental quantifications*.*

Although the dynamic behaviors of the photosynthetic outputs in the circadian clock pathway are the focus of this paper, different photoperiods would affect the periodic characteristics of the circadian clock, so the parameters could be optimized by adding the characteristics of the circadian clock. The optimal cost function is defined by


$$ {\delta}^{\prime }=\sum_{i=G^{\prime }}{\left({y}_i-\overline{y_i}\right)}^2+\sum_{i=G^{\prime }}{\left({\phi}_i-{\phi}_{i_0}\right)}^2+\sum_{i=G^{\prime }}{\left({PI}_i-{PI}_{i_0}\right)}^2:= {\delta}_{SSE}+{\delta}_{\phi }+{\delta}_{PI} $$

where ${G}^{\prime }$ additionally denotes the clock genes *CCA1*/*LHY*, *PRR9*/*PRR7*, *PRR5*/*TOC1*, and *ELF4I/LUX*. The cost function is defined as a sum of three terms. Each term is described in turn as follows: ${\delta}_{SSE}$ measures the difference between the model-simulated expression and the experimental expression; ${\delta}_{\phi }$ measures the differences between the respective simulated peak times (phase) and experimentally observed data; and ${\delta}_{PI}$ is the difference between the period of the oscillation predicted by the model and the experimental period of mRNA levels of genes.

### Photosynthetic parameter detection

The photosynthetic parameters of tea leaves, including net photosynthetic rate (*P*_n_), stomatal conductivity (*G*_s_), intercellular CO_2_ concentration (*C*_i_), and transpiration rate (*T*_r_), were determined by an LI-6400XT portable photosynthesis system (LI-COR, Lincoln, Nebraska, USA) under four different photoperiod treatments. The measured conditions were air flow rate of 500 μmol s^−1^, light intensity of 600 μm^−2^ s^−1^, temperature of (20 ± 1) °C, relative humidity of 70% ~ 75%, and CO_2_ concentration of 400 μmol mol^−1^. Three biological replicates were performed to calculate the mean ± standard error value.

### Determination of leaf stomata

The observation and measurement of tea leaf stomatal opening was an integral part of the tea seedling investigation. To gather precise data, we initially sampled the second leaf from the top of the tea seedling. Before the sample was taken, the dust on the blade surface was thoroughly cleaned to avoid interfering with later observations and measurements. Next, clear nail polish was applied equally to the backs of the leaves. The goal of the process was to increase the transparency of the leaf without destroying the leaf structure, allowing the stomatal structure to be seen more clearly under the microscope. After this application, the tea leaves were allowed to dry naturally for around 5 min. Once the sample was dry, it was secured to a slide using Scotch Tape for microscopic observation. The sample was stored and the biological repetition was repeated three times. After sample preparation, we used an optical microscope (Olympus BX-53, Olympus, Japan). Stomatal opening degree (*c*) was calculated by the formula *c* = π*ab*, where *a* represents half of the length of the stoma and *b* represents half of the width of the stoma [60].

### RNA extraction and RT–qPCR analysis

Total RNA was extracted from tea leaves using a reference RNA extraction kit (RNAsimple Total RNA Kit, Tiangen, Beijing, China). The concentration of RNA samples was determined using a microscopic ultraviolet detector (Nanodrop ND-1000, Thermo Fisher Scientific, USA). The RNA quality was determined by 1.2% agarose gel electrophoresis. The photosynthesis-related gene expression levels associated with the circadian rhythm of the plants was determined by quantitative real-time PCR (RT–qPCR). The detection primer was designed by Primer Remer 6.0 software (Supplementary Data Table S4). RT–qPCR was performed on the Bio-Rad IQ5 fluorescence quantitative PCR platform using the SYBR Premix Ex Taq kit (TaKaRa, Dalian, China). Total RNA extracted from tea plants was reverse-transcribed into cDNA using a reverse transcription kit (TaKaRa, Dalian, China). The *CsGAPDH* gene was selected as the internal reference gene for RT–qPCR analysis, and the amplification primers were CSGAPDH-F and CSGAPDH-R [31]. cDNA of ‘Baiye 1’ was selected under different photoperiods. The amplification system consisted of 10 μl SYBR Green I mix, 0.4 μl forward and reverse fluorescence quantitative primers, 2.0 μl cDNA, and 7.2 μl ddH_2_O (total 20 μl). The amplification procedure was set for denaturation at 95°C for 5 min, denaturation at 95°C for 10 s, annealing at 54°C for 30 s, and extension at 65°C for 15 s, for a total of 40 cycles. The final concentration of primers in the reaction mixture was 0.2 μM. Three biological replicates were performed, and 2^−ΔΔCt^ was used to calculate the relative gene expression levels.

### Numerical simulation and statistical analysis

The numerical simulation of our ordinary differential equations (ODEs) was performed using Python (version 3.10.4), a freely downloadable programming language that can be found on the official Python website (https://www.python.org/downloads/). In Python, we used the classical fourth-order Runge–Kutta method to solve the numerical solution of ODEs. In addition, the rhythmic testing, peak time, and period calculations of the time series datasets were performed using BioDare2, a free online biometric data analysis tool that users could access and use through its official website (https://biodare2.ed.ac.uk/) [61]. The significance level was indicated by an asterisk or a different letter. Microsoft Excel 2019 software was used for data sorting, the significance of differences was analyzed using IBM SPSS Statistics 25.0 (version 25.0), and Duncan multiple comparison method was used to analyze the significance of the difference between the data at the 0.05 level (*P* < 0.05). Finally, all the charts were drawn using Python (version 3.10.4).

## Supplementary Material

Web_Material_uhae226

## Date availability

Data are available in figures of the article and its supplementary materials.
