# Supplementary material for: Interference of skeleton photoperiod in circadian clock and photosynthetic efficiency of tea plant: in-depth analysis of mathematical model
Source: Hortic Res. 2024 Aug 8;11(10):uhae226. doi: 10.1093/hr/uhae226 (PMC11480659; doi:10.1093/hr/uhae226)
Supplement: Web_Material_uhae226 [file web_material_uhae226.zip › 20240726 tea clock hzh HR2 sub clean sub 22.docx]

**Supplementary data**

**
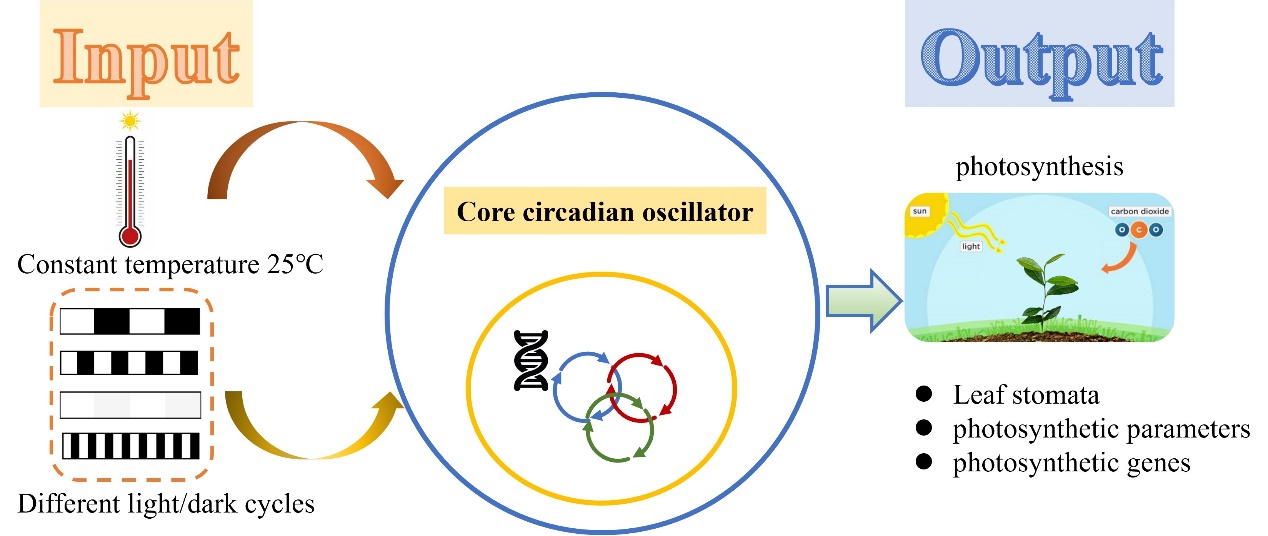
**

**Figure S1**. Flow chart of tea plant circadian rhythm under different photoperiods in tea plant. Constant temperature 25 ^o^C, four photoperiodic treatments, the grey bands represented objective darkness.


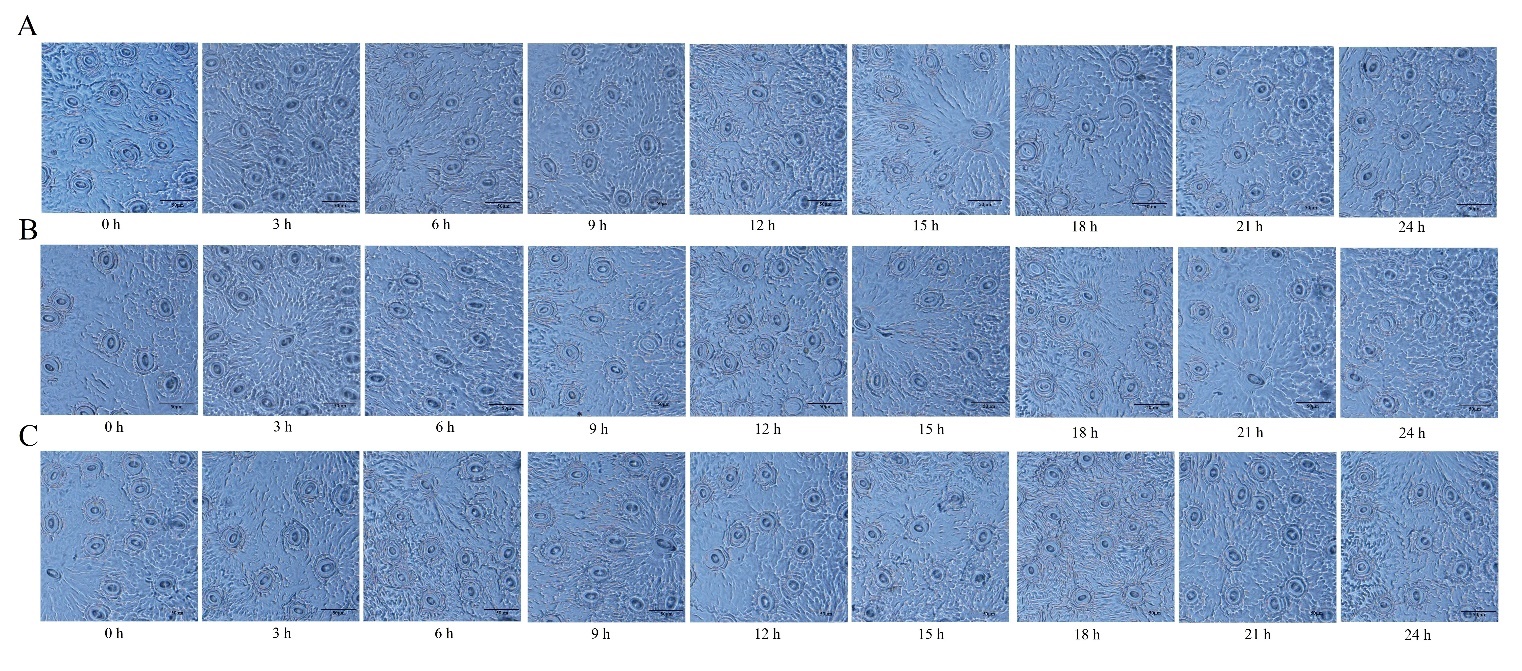


**Figure S2.** Analysis on the circadian rhythm of stomatal opening in tea plants under different photoperiod conditions.

(A) ZT0-ZT12 was daytime and ZT12-ZT24 was nighttime under long day (12L12D). (B) ZT0-ZT6, ZT12-ZT18 were daytime and ZT6-ZT12, ZT18-ZT24 were nighttime under long skeleton photoperiod (6L6D). (C) ZT0-ZT24 was daytime under constant light (24L). ZT indicates zeitgeber time, the time point of light intensity treatment. The stomatal opening of 8~15 stomata randomly selected were recorded. The different time points represent the processing time in the day. Scale bar =50 μm.


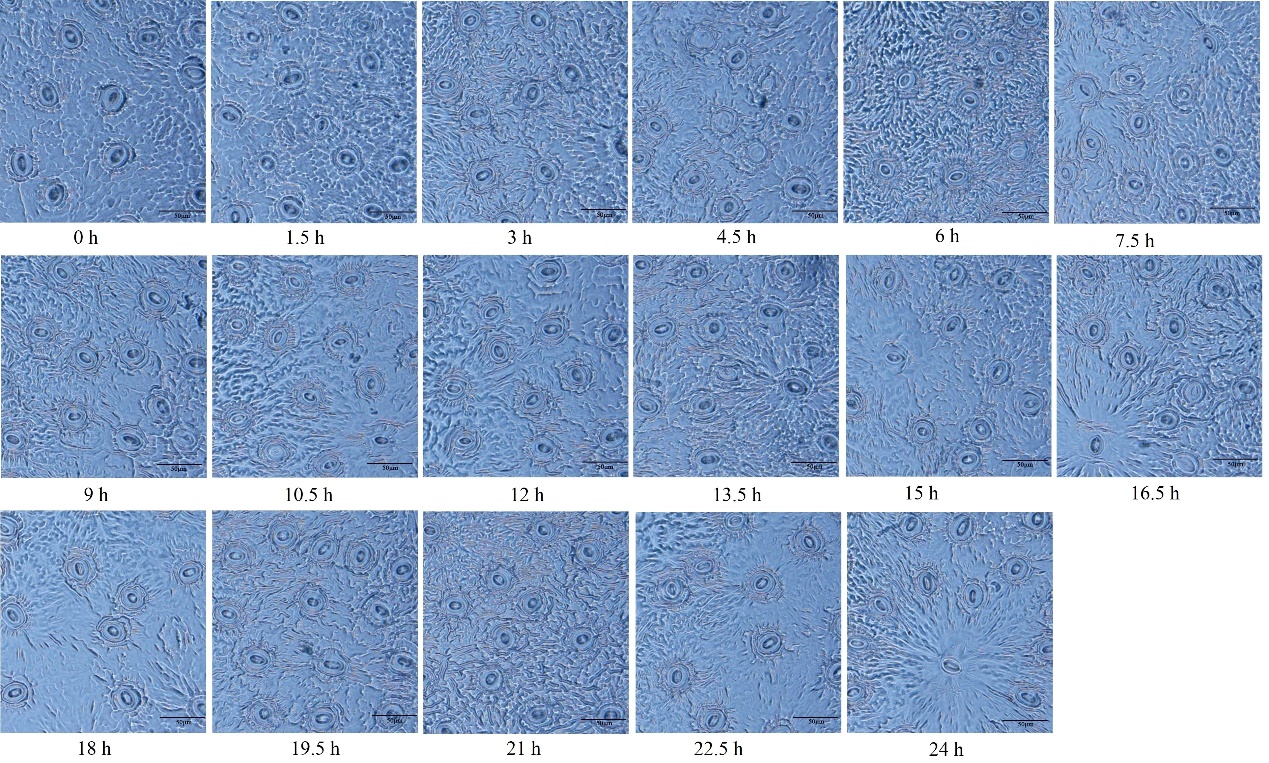


**Figure S3.** Analysis on the circadian rhythm of stomatal opening in tea plants under 3L3D cycles. ZT0-ZT3, ZT6-ZT9, ZT12-ZT15, ZT18-ZT21 were daytime and ZT3-ZT6, ZT9-ZT12, ZT15-ZT18, ZT21-ZT24 were nighttime under 3L3D cycle. The stomatal opening of 8~15 stomata randomly selected were recorded. The different time points represent the processing time in the day. Scale bar =50 μm.

**Table S1.** The length and width of the stomata of tea plant at different times

| Time/h | Stomatal transverse diameter/μm  (12L12D) | Stomatal  longitudinal diameter/μm  (12L12D) | Stomatal transverse diameter/μm  (6L6D) | Stomatal  longitudinal diameter/μm  (6L6D) | Stomatal transverse diameter/μm  (24L) | Stomatal  longitudinal diameter/μm  (24L) | Stomatal transverse diameter/μm  (3L3D) | Stomatal  longitudinal diameter/μm  (3L3D) |
| --- | --- | --- | --- | --- | --- | --- | --- | --- |
| 0 | 9.83±0.23c | 5.99±0.11b | 9.92±0.13c | 5.80±0.13b | 9.87±0.08e | 5.60±0.13cd | 9.92±0.13d | 5.60±0.13cd |
| 1.5 |  |  |  |  |  |  | 10.45±0.08bc | 5.87±0.08bc |
| 3 | 11.41±0.08a | 6.35±0.15ab | 11.20±0.13b | 6.63±0.25a | 10.56±0.13d | 6.67±0.08a | 11.09±0.08a | 6.45±0.08a |
| 4.5 |  |  |  |  |  |  | 10.51±0.08b | 5.97±0.08bc |
| 6 | 11.52±0.26a | 6.51±0.20a | 12.48±0.13a | 5.80±0.26b | 11.52±0.13a | 6.51±0.20ab | 9.81±0.08de | 5.71±0.08c |
| 7.5 |  |  |  |  |  |  | 10.19±0.20c | 5.76±0.13c |
| 9 | 10.88±0.13b | 6.40±0.13ab | 9.81±0.13c | 5.01±0.20d | 11.20±0.13b | 6.40±0.13b | 10.56±0.13b | 6.08±0.13b |
| 10.5 |  |  |  |  |  |  | 9.76±0.13de | 5.12±0.13e |
| 12 | 11.25±0.15ab | 6.29±0.08ab | 9.87±0.13c | 4.96±0.20d | 10.61±0.20d | 6.29±0.20b | 9.76±0.13de | 5.28±0.13de |
| 13.5 |  |  |  |  |  |  | 10.40±0.13bc | 5.71±0.08c |
| 15 | 9.65±0.08cd | 4.52±0.26c | 11.36±0.13b | 4.96±0.13d | 10.88±0.13c | 5.76±0.13c | 10.15±0.09c | 5.75±0.18c |
| 16.5 |  |  |  |  |  |  | 10.13±0.08c | 5.60±0.13cd |
| 18 | 9.60±0.13cd | 4.68±0.13c | 11.04±0.13b | 4.96±0.13d | 10.60±0.18d | 5.48±0.03d | 9.60±0.13e | 5.33±0.20de |
| 19.5 |  |  |  |  |  |  | 10.13±0.08c | 5.60±0.11cd |
| 21 | 9.25±0.16d | 5.75±0.14b | 9.76±0.13c | 5.44±0.13c | 10.45±0.27de | 6.61±0.08a | 9.61±0.08e | 6.03±0.08b |
| 22.5 |  |  |  |  |  |  | 9.76±0.13de | 5.12±0.13e |
| 24 | 9.93±0.14c | 5.87±0.08b | 9.76±0.13c | 5.71±0.13b | 10.72±0.13cd | 6.24±0.13bc | 10.03±0.08cd | 5.44±0.13d |

**Table S2.** Core variables for tea plant circadian clock effect on photosynthesis under light entrainment.

The first column lists the sequence of variables and the definition of each variable is given in the second column.

| Symbol | Definition |
| --- | --- |
| $\boldsymbol{[}\mathbf{MCL}\boldsymbol{]}$ | concentration of CCA1/LHY mRNA |
| $\boldsymbol{[}\mathbf{CL}\boldsymbol{]}$ | concentration of CCA1/LHY protein |
| $\boldsymbol{[}\mathbf{MP97}\boldsymbol{]}$ | concentration of PRR9/PRR7 mRNA |
| $\boldsymbol{[}\mathbf{P97}\boldsymbol{]}$ | concentration of PRR9/PRR7 protein |
| $\boldsymbol{[}\mathbf{MP51}\boldsymbol{]}$ | concentration of PRR5/TOC1 mRNA |
| $\boldsymbol{[}\mathbf{P51}\boldsymbol{]}$ | concentration of PRR5/TOC1 protein |
| $\boldsymbol{[}\mathbf{MEL}\boldsymbol{]}$ | concentration of ELF4/LUX mRNA |
| $\boldsymbol{[}\mathbf{EL}\boldsymbol{]}$ | concentration of ELF4/LUX protein |
| $\boldsymbol{P}$ | The activation of light-sensitive proteins |
| $\boldsymbol{[}\mathbf{MLhcb1}\boldsymbol{]}$ | the concentration of Lhcb1 mRNA |
| $\boldsymbol{[}\mathbf{Lhcb1}\boldsymbol{]}$ | the concentration of Lhcb1 protein |
| $\boldsymbol{[}\mathbf{MRbcS1}\boldsymbol{]}$ | the concentration of $\mathbf{RbcS1}$ mRNA |
| $\boldsymbol{[}\mathbf{RbcS1}\boldsymbol{]}$ | the concentration of $\mathbf{RbcS1}$ protein |
| $\boldsymbol{[}\mathbf{MatpA}\boldsymbol{]}$ | the concentration of $\mathbf{atpA}$ mRNA |
| $\boldsymbol{[}\mathbf{atpA}\boldsymbol{]}$ | the concentration of $\mathbf{atpA}$ protein |

**Table S3.** The basic parameters of tea circadian clock model under constant light.

| Parameters | Desceiption | Values(unit) |
| --- | --- | --- |
| $\boldsymbol{v}_{\boldsymbol{1}}$ | *CCA1/LHY* synthesis | 1.09 nM h^-1^ |
| $\boldsymbol{v}_{\boldsymbol{1}\boldsymbol{L}}$ | *CCA1/LHY* light-induced synthesis | 0.714 nM h^-1^ |
| $\boldsymbol{v}_{\boldsymbol{1}\boldsymbol{A}}$ | *CCA1/LHY* synthesis induced by LWD1 | 1.89 nM h^-1^ |
| $\boldsymbol{v}_{\boldsymbol{2}\boldsymbol{A}}$ | *PRR9/PRR7* synthesis | 0.71 nM h^-1^ |
| $\boldsymbol{v}_{\boldsymbol{2}\boldsymbol{L}}$ | *PRR9/PRR7* light-induced synthesis | 2.78 nM h^-1^ |
| $\boldsymbol{v}_{\boldsymbol{2}\boldsymbol{B}}$ | *PRR9/PRR7* synthesis induced by LWD1 | 1.9 nM h^-1^ |
| $\boldsymbol{v}_{\boldsymbol{3}}$ | *PRR5/TOC1* synthesis induced by RVE8/LNK1 | 1.0 nM h^-1^ |
| $\boldsymbol{v}_{\boldsymbol{3}\boldsymbol{B}}$ | *PRR5/TOC1* synthesis induced by LWD1 | 2.2 nM h^-1^ |
| $\boldsymbol{v}_{\boldsymbol{4}}$ | *ELF4/LUX* synthesis | 0.2 nM h^-1^ |
| $\boldsymbol{v}_{\boldsymbol{4}\boldsymbol{L}}$ | *ELF4/LUX* light-induced synthesis | 2.94 nM h^-1^ |
| $\boldsymbol{k}_{\boldsymbol{1}\boldsymbol{L}}$ | *CCA1/LHY* mRNA degradation (light) | 0.53 h^-1^ |
| $\boldsymbol{k}_{\boldsymbol{1}\boldsymbol{D}}$ | *CCA1/LHY* mRNA degradation (dark) | 0.21 h^-1^ |
| $\boldsymbol{k}_{\boldsymbol{2}}$ | *PRR9/PRR7* mRNA degradation | 0.35 h^-1^ |
| $\boldsymbol{k}_{\boldsymbol{3}}$ | *PRR5/TOC1* mRNA degradation | 0.56 h^-1^ |
| $\boldsymbol{k}_{\boldsymbol{4}}$ | *ELF4/LUX* mRNA degradation | 0.27 h^-1^ |
| $\boldsymbol{p}_{\boldsymbol{1}}$ | CCA1/LHY translation | 0.76 h^-1^ |
| $\boldsymbol{p}_{\boldsymbol{1}\boldsymbol{L}}$ | CCA1/LHY light-induced translation | 0.42h^-1^ |
| $\boldsymbol{p}_{\boldsymbol{2}}$ | PRR9/PRR7 translation | 1.01 h^-1^ |
| $\boldsymbol{p}_{\boldsymbol{3}}$ | PRR5/TOC1 translation | 0.64 h^-1^ |
| $\boldsymbol{p}_{\boldsymbol{4}}$ | ELF4/LUX translation | 1.01 h^-1^ |
| $\boldsymbol{d}_{\boldsymbol{1}}$ | CCA1/LHY degradation | 0.68 h^-1^ |
| $\boldsymbol{d}_{\boldsymbol{2}\boldsymbol{D}}$ | PRR9/PRR7 degradation (dark) | 1.05 h^-1^ |
| $\boldsymbol{d}_{\boldsymbol{2}\boldsymbol{L}}$ | PRR9/PRR7 degradation (light) | 0.59 h^-1^ |
| $\boldsymbol{d}_{\boldsymbol{3}\boldsymbol{D}}$ | PRR5/TOC1 degradation (dark) | 0.48 h^-1^ |
| $\boldsymbol{d}_{\boldsymbol{3}\boldsymbol{L}}$ | PRR5/TOC1 degradation (light) | 0.78 h^-1^ |
| $\boldsymbol{d}_{\boldsymbol{4}\boldsymbol{D}}$ | ELF4/LUX degradation (dark) | 1.21 h^-1^ |
| $\boldsymbol{d}_{\boldsymbol{4}\boldsymbol{L}}$ | ELF4/LUX degradation (light) | 0.28 h^-1^ |
| $\boldsymbol{K}_{\boldsymbol{0}}$ | Inhibition of CCA1/LHY by CCA1/LHY | 5.07 nM |
| $\boldsymbol{K}_{\boldsymbol{1}}$ | Inhibition of CCA1/LHY by PRR9/PRR7 | 0.16 nM |
| $\boldsymbol{K}_{\boldsymbol{2}}$ | Activation of CCA1/LHY by LWD1 | 1.18 nM |
| $\boldsymbol{K}_{\boldsymbol{3}}$ | Inhibition of PRR9/PRR7 by PRR5/TOC1 | 1.2 nM |
| $\boldsymbol{K}_{\boldsymbol{4}}$ | Inhibition of PRR9/PRR7 by ELF4/LUX | 0.4 nM |
| $\boldsymbol{K}_{\boldsymbol{5}}$ | Inhibition of PRR9/PRR7 by CCA1/LHY | 0.62 nM |
| $\boldsymbol{K}_{\boldsymbol{5}\boldsymbol{b}}$ | Activation of PRR9/PRR7 by LWD1 | 4.1 nM |
| $\boldsymbol{K}_{\boldsymbol{6}}$ | Activation of PRR5/TOC1 by LWD1 | 0.46 nM |
| $\boldsymbol{K}_{\boldsymbol{7}}$ | Inhibition of PRR5/TOC1 by PRR5/TOC1 | 2.0 nM |
| $\boldsymbol{K}_{\boldsymbol{7}\boldsymbol{a}}$ | Activation of PRR5/TOC1 by RVE8/LNK1 | 0.5 nM |
| $\boldsymbol{K}_{\boldsymbol{8}}$ | Inhibition of ELF4/LUX by CCA1/LHY | 0.36 nM |
| $\boldsymbol{K}_{\boldsymbol{9}}$ | Inhibition of ELF4/LUX by PRR5/TOC1 | 1.9 nM |
| $\boldsymbol{K}_{\boldsymbol{10}}$ | Inhibition of ELF4/LUX by ELF4/LUX | 1.9 nM |

**Table S4.** The basic parameters of tea photosynthetic genes controlled by circadian clock under constant light.

| Parameters | Desceiption | Values(unit) |
| --- | --- | --- |
| $\boldsymbol{v}_{\boldsymbol{5}}$ | *Lhcb1* synthesis | 12.6 nM h^-1^ |
| $\boldsymbol{v}_{\boldsymbol{6}}$ | *RbcS1* synthesis | 6.7 nM h^-1^ |
| $\boldsymbol{v}_{\boldsymbol{7}}$ | *atpA* synthesis | 9.6 nM h^-1^ |
| $\boldsymbol{k}_{\boldsymbol{5}}$ | *Lhcb1* mRNA degradation | 1.6 h^-1^ |
| $\boldsymbol{k}_{\boldsymbol{6}}$ | *RbcS1* mRNA degradation | 0.25 h^-1^ |
| $\boldsymbol{k}_{\boldsymbol{7}}$ | *atpA* mRNA degradation | 0.13 h^-1^ |
| $\boldsymbol{p}_{\boldsymbol{5}}$ | Lhcb1 translation | 0.68 h^-1^ |
| $\boldsymbol{p}_{\boldsymbol{6}}$ | RbcS1 translation | 0.56 h^-1^ |
| $\boldsymbol{p}_{\boldsymbol{7}}$ | atpA translation | 0.16 h^-1^ |
| $\boldsymbol{d}_{\boldsymbol{5}}$ | Lhcb1degradation | 0.86 h^-1^ |
| $\boldsymbol{d}_{\boldsymbol{6}}$ | RbcS1 degradation | 0.46 h^-1^ |
| $\boldsymbol{d}_{\boldsymbol{7}}$ | atpA degradation | 0.48 h^-1^ |
| $\boldsymbol{K}_{\boldsymbol{11}}$ | Activation of Lhcb1 by CCA1/LHY | 1.5 nM |
| $\boldsymbol{K}_{\boldsymbol{12}}$ | Inhibition of Lhcb1by PRR5/TOC1 | 0.2 nM |
| $\boldsymbol{K}_{\boldsymbol{13}}$ | Inhibition of RbcS1by CCA1/LHY | 1.5 nM |
| $\boldsymbol{K}_{\boldsymbol{14}}$ | Inhibition of atpA by CCA1/LHY | 1.12 nM |

**Table S5.** Primers for RT-qPCR.

| Gene | Forward primer sequence (5'-3') | Reverse primer sequence (5'→3') |
| --- | --- | --- |
| *CsCCA1* | GCTATTGCTGCTGCTACA | CTCTACATCACTACTGGAAGG |
| *CsPRR9* | AGGCAGAGAAGAACAAGAAT | TCAAGGTAAGAAGAGCGAAT |
| *CsPRR5* | TTAGTGACGAAGGTGTGAAT | TAAGAAGAGCGAATCCAGAG |
| *CsELF4* | CAATCACCTCCATCTCCAA | GCTGTCTTCGTCTTCCTTA |
| *CsLhcb1* | GGCAGAGGAAGGATCAGCAT | CACGGTTCTTGGCGAATGTT |
| *CsatpA* | TGAAGTAATGGCAGGTGAAT | AAGAGGCTCATATACGGAAC |
| *CsRbcS1* | TCCAACAATGACATCACTTC | GAATCCGTGCTCAGTCTC |
| *GAPDH* | TTGGCATCGTTGAGGGTCT | CAGTGGGAACACGGAAAGC |

**Supplementary Material File:**

**Circadian clock model in tea plant under different photoperiods**

Drawing from the initial computational model for circadian-clock-regulated photosynthesis in tea plants, we constructed a light-entrainment model comprising four sets of circadian genes. This integrated model incorporates key photosynthesis-related genes (*Lhcb1*, *psbA*, *atpA*) and outputs variables. **Figure 4** depicted the gene regulation diagram of the model, while the model itself comprises a set of ordinary differential equations detailed in equations (S1) through (S15).

The temporal evolution of mRNA and protein levels of variables involved in the circadian clock, such as CL (CCA1/LHY), P97 (PRR9/PRR7), P51 (PRR5/TOC1), EL (ELF4/LUX), and P activity, was governed by the following set of differential equations:

$\frac{\text{d}[\mathrm{MCL}]}{\text{d}t}=\left( v_{1}+v_{1L}L\left( t \right)\left[ P \right]+v_{1A}\frac{\left( \frac{\left[ \mathrm{LWD} \right]}{K_{2a}} \right)^{2}}{1+\left( \frac{\left[ \mathrm{LWD} \right]}{K_{2a}} \right)^{2}} \right)\frac{1}{1+\left( \frac{\left[ \mathrm{CL} \right]}{K_{0}} \right)^{2}+\left( \frac{\left[ P97 \right]}{K_{1}} \right)^{2}+\left( \frac{\left[ P51 \right]}{K_{2}} \right)^{2}}-(k_{1L}L\left( t \right)+k_{1D}D\left( t \right))[\mathrm{MCL}]$ (S1)

$\frac{\text{d}[\mathrm{CL}]}{\text{d}t}=(p_{1}+p_{1L}L\left( t \right))[\mathrm{MCL}]-d_{1}[\mathrm{CL}]$ (S2)

$\frac{d[MP97]}{dt}=\left( v_{2L}L\left( t \right)[P]+v_{2A}+v_{2B}\frac{\left( \frac{\left[ \mathrm{LWD} \right]}{K_{5b}} \right)^{2}}{1+\left( \frac{\left[ \mathrm{LWD} \right]}{K_{5b}} \right)^{2}} \right)\frac{1}{1+\left( \frac{\left[ P51 \right]}{K_{3}} \right)^{2}+\left( \frac{\left[ \mathrm{EL} \right]}{K_{4}} \right)^{2}+\left( \frac{\left[ \mathrm{CL} \right]}{K_{5}} \right)^{2}}-k_{2}[MP97]$ (S3)

$\frac{\text{d}[P97]}{\text{d}t}=p_{2}[MP97]-(d_{2D}D\left( t \right)+d_{2L}L\left( t \right))[P97]$  (S4)

$\frac{d[MP51]}{dt}=\left( v_{3A}+v_{3B}\frac{\left( \frac{\left[ \mathrm{LWD} \right]}{K_{7a}} \right)^{2}}{1+\left( \frac{\left[ \mathrm{LWD} \right]}{K_{7a}} \right)^{2}} \right)\frac{1}{1+\left( \frac{\left[ \mathrm{CL} \right]}{K_{6}} \right)^{2}+\left( \frac{\left[ P51 \right]}{K_{7}} \right)^{2}}-k_{3}[MP51]$ (S5)

$\frac{\text{d}[P51]}{\text{d}t}=p_{3}\left[ MP51 \right]-(d_{3D}D\left( t \right)+d_{3L}L\left( t \right))[P51]$ (S6)

$\frac{d[\mathrm{MEL}]}{dt}=(v_{4}+v_{4L}L\left( t \right)[P])\frac{1}{1+\left( \frac{\left[ \mathrm{CL} \right]}{K_{8}} \right)^{2}+\left( \frac{\left[ P51 \right]}{K_{9}} \right)^{2}+\left( \frac{\left[ \mathrm{EL} \right]}{K_{10}} \right)^{2}}-k_{4}[\mathrm{MEL}]$ (S7)

$\frac{\text{d}[\mathrm{EL}]}{\text{d}t}=p_{4}[\mathrm{MEL}]-(d_{4D}D\left( t \right)+d_{4L}L\left( t \right))[\mathrm{EL}]$ (S8)

$\frac{\text{d}[P]}{\text{d}t}=p_{12}\left( 1-[P] \right)D\left( t \right)-d_{12}[P]L\left( t \right)$ (S9)

Since the function of LWD1 is periodic, the concentration of LWD1 is assumed as a sine function.

The functions L, D represent light and darkness, respectively. L=1 and D=0 when the system is in light, whereas L=0 and D=1 when the system is in dark.

The dynamics of mRNA and protein levels for photosynthesis-response genes *Lhcb1*, *RbcS1*, and *atpA* were modeled as follows:

$\frac{\text{d}[MLhcb1]}{\text{d}t}=v_{5}\frac{\left( \frac{\left[ \mathrm{CL} \right]}{K_{11}} \right)^{2}}{1+\left( \frac{\left[ \mathrm{CL} \right]}{K_{11}} \right)^{2}}\cdot\frac{1}{1+\left( \frac{\left[ P51 \right]}{K_{12}} \right)^{2}}-k_{5}[MLhcb1]$ (S10)

$\frac{\text{d}[Lhcb1]}{\text{d}t}=p_{5}[MLhcb1]-d_{5}[Lhcb1]$ (S11)

$\frac{\text{d}[MRbcS1]}{\text{d}t}=v_{6}\frac{1}{1+\left( \frac{\left[ \mathrm{CL} \right]}{K_{13}} \right)^{2}}-k_{6}[MRbcS1]$ (S12)

$\frac{\text{d}[RbcS1]}{\text{d}t}=p_{6}[MRbcS1]-d_{6}[RbcS1]$ (S13)

$\frac{\text{d}[\mathrm{MatpA}]}{\text{d}t}=v_{7}\frac{1}{1+\left( \frac{\left[ \mathrm{CL} \right]}{K_{14}} \right)^{2}}-k_{7}[\mathrm{MatpA}]$ (S14)

$\frac{\text{d}[\mathrm{atpA}]}{\text{d}t}=p_{7}[\mathrm{MatpA}]-d_{7}[\mathrm{atpA}]$ (S15)
